# The experiences of teleradiology end users regarding role extension in a rural district of the North West province: A qualitative analysis

**DOI:** 10.4102/phcfm.v12i1.2227

**Published:** 2020-03-19

**Authors:** Hafsa Essop, Mable Kekana

**Affiliations:** 1 Department of Radiography, Faculty of Health Sciences, University of Pretoria, Pretoria, South Africa

**Keywords:** teleradiology, district hospital, radiographer, referring clinician, radiologist, capacity building, role extension

## Abstract

**Background:**

Teleradiology was implemented across South Africa, to provide reporting services to rural healthcare institutes without a radiologist. This is guided by standard operating procedure manuals (SOP) which standardise the quality of services provided. From observation, end users, namely, the radiographer and referring clinician, experience challenges in fulfilling the roles extending beyond the SOP.

**Aim:**

To explore the end users’ experiences within this context and the impact it has on service delivery.

**Setting:**

A rural district in North West province, South Africa.

**Method:**

This was a qualitative, exploratory, descriptive study. Focus group discussions were held with radiographers and referring clinicians from the teleradiology site in the North West province. A one-on-one interview was conducted with a private radiologist at the reporting site in Gauteng. An interview guide was used to ask open-ended questions to address the aim of the study.

**Results:**

At the teleradiology site, radiographers and referring clinicians are performing extended roles, not described in the teleradiology service-level agreement (SLA) and felt poorly equipped to fulfil these roles. They also felt that the private radiologists needed training on interprofessional collaboration to understand the challenges facing health professionals at these rural sites.

**Conclusion:**

SLA’s should align with the clinical needs and practices of the district. This should guide the specific training needs of the end users practicing in rural areas, to support their extended roles in the teleradiology setting. Training should be in-house, ongoing and consistent to cater for the influx of health professionals entering the rural setting using teleradiology systems.

## Introduction

The National Development Plan (NDP) of South Africa 2030 refers to providing access to healthcare and ensuring that this care is of optimal quality, particularly in rural communities.^1^ Unfortunately, the quality of care in the South African primary healthcare setting is an area of concern, where care has been described as complaisant and of poor quality.^2,3^ For this reason, the National Department of Health (DoH) has adopted eHealth strategies to improve the provision of healthcare services at the district level. District hospitals receive referrals from clinics and community healthcare centres (CHC) and provide patients coming from these centres with further diagnostic and therapeutic support by general healthcare practitioners.^4^ Teleradiology, a subcategory of telemedicine, aims to provide specialised radiological services, such as radiological reports, to remote rural communities that do not have access to radiologists.^5^

Teleradiology in South Africa was first implemented in 1999, across three provinces, namely, Free State, Mpumalanga and the North West.^6,7,8^ Many developments have since occurred, with the addition of more teleradiology sites. In KwaZulu-Natal, South Africa, teleradiology successfully facilitates appropriate patient management at the district hospital level, reducing unnecessary transfers to urban tertiary institutes.^6^ Other South African provinces, including the North West, Mpumalanga and Free State, also benefit from teleradiology; however, experiences with current systems have not been documented. Teleradiology plays a significant role in the primary healthcare setting in South Africa, but the quality of care through the teleradiology medium remains unexplored.

### Background

This study focusses on teleradiology in the North West province of South Africa, as there has been limited exploration of the system’s effectiveness following the first report in 2001. The vision of the North West Department of Health (NW DoH) for teleradiology is to provide access to specialised imaging procedures, reports on all computed tomography (CT) examinations, radiographs and mammograms, specialist consultations with doctors and continuous professional development presentations.^9^ To gain an understanding of the foundation of teleradiology applications in the North West province, the researcher provides a brief overview of the first eHealth implementations in the North West province, where the study was conducted.

Teleradiology was piloted in the North West province for the first time in 1999, with the main objective of providing specialised radiological reporting to rural areas that were non-existent before this implementation.^6^ Three teleradiology sites were initially installed across the province. In 2000, the Medical Research Council in conjunction with the National DoH evaluated the progress of the first phase of teleradiology implementation. The report revealed both challenges and achievements of the teleradiology systems from the end users’ perspective.^6^ Following this evaluation, many developments were noted in this province. These included the addition of two more teleradiology sites, a new private radiology service provider and the appointment of new health professionals, including radiographers, referring clinicians and radiologists.

**FIGURE 1:**
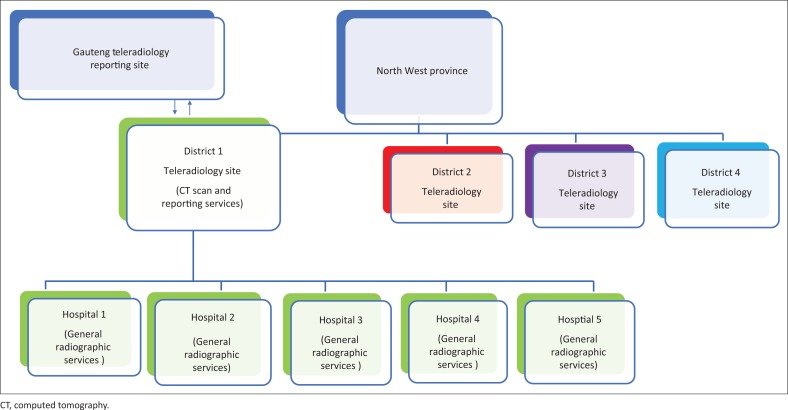
Teleradiology sites in district hospitals in the North West province.

**FIGURE 2:**
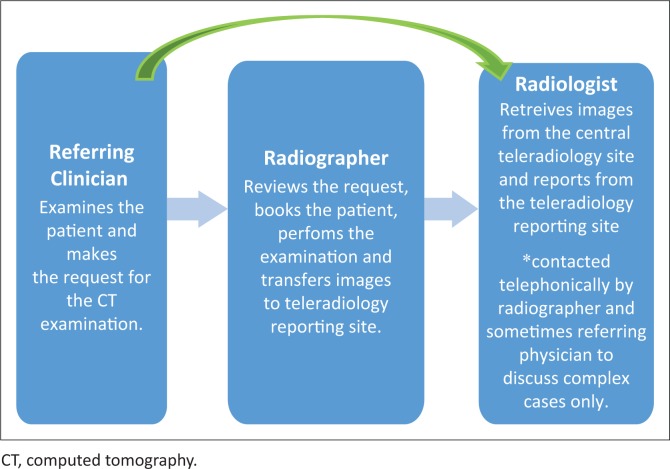
Teleradiology workflow in district 1.

The North West province is divided into four main districts (Figure 1). Teleradiology is implemented in all four districts, mainly to assist the onsite radiologist with reporting of CT scan examinations (districts 2, 3 and 4 – Figure 2). In this study, the researcher will focus on district 1, comprising the central teleradiology site, which has a CT scanner that provides services to its own hospital and five satellite district hospitals. This district was chosen for this study because it does not have onsite radiologists as with other districts in the North West province, and therefore fully relies on teleradiology to provide radiological reporting services. The central teleradiology site of district 1 does not have a radiology nurse and offers general radiographic services, ultrasound and CT. The satellite hospitals of district 1 are also district hospitals; however, they do not offer specialised CT examinations and therefore refer patients to the central teleradiology site for further diagnostic work-up using CT scan. Physicians at this central teleradiology site are called to inject contrast media for their own patients and for patients being referred by other physicians from the five satellite hospitals. Teleradiology is only used for reporting CT examinations and excludes mammography and general radiographs, which are offered at other sites within the province. Radiographers at the central teleradiology site perform CT examinations independently, without the direct guidance from the radiologist. The images are then sent through the teleradiology system to the radiologists located at the teleradiology reporting site in Gauteng, who report and send back the images to the central site. Radiographers then access the reports and send to the referring physicians from satellite sites. All these health professionals who utilise the teleradiology system to provide a service for the patient are referred to as ‘end users’.

In 2013, the NW DoH issued a new standard operating procedure (SOP) manual and service-level agreement (SLA) to all teleradiology sites within the province, to ensure standardisation of all teleradiology practices in all districts. The SOP defines the scope of practice for each of the healthcare professionals involved in the teleradiology operation, namely, the radiographer, nurse, physician and radiologist^9^:

1. Radiographers must:
  - ensure that they produce quality images at all times
  - distribute radiological examination reports to the relevant medical officer as soon as possible
  - monitor turnaround time of reports and intervene where there are challenges
  - make sure that duplicate reports are kept in a file in the radiology department
  - report utilisation of the service on a monthly basis
  - monitor expenditure of the project per site.
2. Nurses shall ensure that all patients undergoing fluoroscopy procedures/CT scan have the necessary preparations as stated in prescribed protocols.
3. Physicians shall ensure that the examination is indeed necessary and that adequate and clear clinical history is provided at all times.
4. Radiologist: the role of the radiologist as designated by the Ionizing Radiation (Medical Exposure) Regulations 2000 (IR (ME) R); in justifying the examination remains paramount and is dependent on the components of each clinical case; shall indicate if routine protocol requires any modification and if the correct type and amount of contrast is injected.

The teleradiology workflow in district 1 significantly differs from the other districts and traditional radiology workflow, whereby the physician would consult with the onsite radiologist to discuss the CT request. Upon approval of the request from the radiologist, the request will be sent to the radiographer who will be guided on the imaging plan by the radiologist, who would further indicate if there is a need for contrast media. However, in district 1, as shown in Figure 2, the physician consults with the radiographer. The physician will only telephonically contact the radiologist (green arrow) when there is uncertainty from the radiographer about the CT request.

From the observations made by the researcher within the context of the teleradiology systems in district 1, that is, before embarking on this research project, it appeared that the onsite end users, namely, radiographers and referring physicians, undertake tasks that are within the scope of the radiologist as stated in the SOP. For this reason, the researchers aimed to explore the views of the end users of this district to ascertain how they have experienced working in this teleradiology environment. The inclusion of the end users from the satellite hospitals was also necessary, as they also engage with the radiographers and radiologists from the central teleradiology site. The objective of the study was therefore to describe the views of the radiographers, the physician (referred to as the referring clinician) and the radiologist, as well as to describe recommendations on how the operation and thus service delivery can be improved. The research question the study attempted to answer was, ‘what are the views of the teleradiology end users regarding their experiences in this teleradiology context guided by the generic teleradiology SOP for the North West province?’

## Negotiation access and consent to participate

Following ethics approval, the researcher contacted the chief executive officer (CEO) of each district hospital and the central teleradiology site in district 1, to inform them of the study to be conducted. An invitation was then sent out to the clinical managers and the heads of the radiography departments inviting each category of health professionals to a focus group interview on different days. An information leaflet was attached to the invitation, explaining the nature and content of the study to assist the end users in deciding on whether or not they would like to participate. The end users were then asked to respond to the researcher within a stipulated time frame, if they were willing to participate, and thus implied consent was given. On the day of the focus group interviews, the researcher provided consent forms which the participants had to complete before commencing. For the private radiology service provider, the researcher scheduled a meeting with the CEO of the company explaining the nature of the study to be conducted. This meeting also ensured that no conflict of interest arose from the study. The radiologist consent form was signed by the radiologist on the day of the interview.

## Methods

### Study design

We used a qualitative approach, with an exploratory descriptive research design to answer the following questions: ‘what are the views of teleradiology end users regarding their experiences of teleradiology?’ and ‘what impact does their experience have on primary healthcare service delivery?’ This research design enabled the researcher to explore and probe deeper into the end user’s experiences.

### Setting

The study was conducted in the North West province in district 1, as described in the background as well as the teleradiology reporting site in Gauteng.

### Study population, size and sampling

The study population included radiographers and physicians from the five satellite hospitals of district 1 as well as the central teleradiology site, which is also a district hospital as shown in Table 1. A purposeful sampling method was used as the researcher invited all the end users described in the study population. The participants who RSVP’d were included in the sample size and are shown in Table 1. At the time of recruiting, the radiology service provider had five radiologists; this number had reduced to three radiologists, and only one was available for an interview. Because of the qualitative, exploratory and descriptive nature of the study, no definitive sample size was established, as the researcher was interested in information-rich descriptions from the participants and not statistical numbers of individuals for the focus group interviews. Liamputtong suggests that qualitative studies should not have too many participants, as this may create the risk of increasing the complexity of the process of.^10^ This author further recommends that 6–12 participants are sufficient for one focus group session. It was, however, important that there was representation from the teleradiology central site as this is where the actual image acquisition and transfer takes place.

**TABLE 1:** Staff complement and total study population of district 1.

District 1	Radiographers	Referring clinicians	Radiologist
Teleradiology central site 1 (North West province)	8	20	0
District hospital 1	6	10	0
District hospital 2	4	10	0
District hospital 3	1	3	0
District hospital 4	3	5	0
District hospital 5	2	5	0
Teleradiology reporting site (Gauteng province)	-	-	5
**Total study population invited**	**24**	**53**	**5**

### Data collection

Two data collection methods were used in this study. The first was two focus group discussions (FGDs), one for the radiographers (six participants) and one for the physicians (12 participants) on separate days. These FGDs were held at the central teleradiology site in the North West province where the images are acquired, as it is centrally located for all participants and comprises mostly healthcare professionals, as shown in Table 1. The researcher herself facilitated the discussions through an open-ended interview guide in English, a common language to all participants. The role of the researcher is twofold: firstly, preparing the interview guide; secondly, conducting the actual interview to generate quality data during the focus group interview. Krueger and Casey state that the quality and quantity of the data collected are largely influenced by the interaction between the researcher and the participants during the FGD.^11^ The researcher’s role as the interviewer is to ensure that the participants do not dwell on irrelevant experiences, but rather lead the participants to the research question, which the researcher can delve deeper into, by means of probing questions.^12^

The interview guide comprised similar open-ended questions for all participants. The responses of the participants guided the probing questions. The questions included in the interview guide were the following:

1. Can you share your experiences of using teleradiology?
2. How does this experience influence the service you deliver?
3. What do you recommend to improve the use of teleradiology?

The second data collection method was a one-on-one interview with the radiologist at the teleradiology reporting site. A date was scheduled for the radiologist’s interview in Gauteng at the private radiology practice where the reporting was done. Initially, the researcher had planned for focus group interviews with the five radiologists described in the study population; however, at the time of data collection, only one radiologist was available, who was interviewed using the same interview guide.

### Data analysis

Audio recordings were transcribed and analysed by means of content analysis. This entails systematic identifying, classifying and coding of themes.^13^ Data were coded using the method described by Zhang and Wildemuth,^14^ which included eight stringent steps in the coding process to develop categories and themes: (1) preparing the data, (2) defining the unit of analysis, (3) developing categories and a coding scheme, (4) testing the coding scheme on a sample of text, (5) coding of all text, (6) assessing coding consistency, (7) drawing conclusions from coded data and (8) reporting methods and findings.

#### Step 1: Preparing the data

The researcher transcribed the audio recording from the three focus group interviews verbatim. Through the transcription process, the researcher had the opportunity to immerse self in the data. This is supported by Liamputtong, and Polit and Beck, who encouraged the qualitative researcher to undertake this process, as a means of becoming familiar with the process of data analysis.^10,15^

#### Step 2: Defining the unit of analysis

A unit of analysis refers to the sample that the researcher aims to analyse and must be established in the preparation phase of content analysis.^16^ Three groups of research participants are taken separately as the units of analysis for this study. The decision to have these three groups as separate units of analysis was taken because each discipline has its own unique experiences in the teleradiology setting. To generalise the unit of analysis as a whole would mean overlooking the significant challenges experienced by each group of end users.

#### Step 3: Developing coding and categorisation scheme

Descriptive codes are formed using terms that are close to the participants’ actual words to best describe their views.^10^ These words form a ‘basic vocabulary’ of data, which will be used to create categories. The descriptive coding method was appropriate for this study, as it aligned well with the study design and objectives.

#### Step 4: Testing the coding scheme on a sample of text

According to Zhang and Wildemuth, once a coding scheme has been formulated, the researcher must ensure its validity before progressing onto further analysis.^14^ Credibility of the study is maintained through member checking or the use of a co-coder. This step was achieved by the researcher coding a small sample of data first, which was verified by the supervisor to check its accuracy before commencing with the full coding. This was done for every focus group interview.

#### Step 5: Coding of all text

When the supervisor was satisfied with the sample coding used, the researcher commenced with coding all the text from each group. The supervisor was actively involved in all stages of the coding process to ensure that the research did not drift into the researcher’s own preconceived ideas and personal experiences within the field of teleradiology.^17^

#### Step 6: Assessing coding consistency

Because of the inductive nature of qualitative studies, the researcher constantly revisited the codes and reflected on the initial analysis.^18^ Creswell supports this strategy and explains that in inductive reasoning the researcher will draw categories and themes from the data.^19^

#### Step 7: Drawing conclusions from the coded data

In this step, the researcher critically analysed the different dimensions presented in the categories to uncover the emerging themes. Themes are described as presenting the data on an interpretive level.^16^

#### Step 8: Reporting methods and findings

This is the last step in the content analysis process, and its aim is to truthfully and explicitly describe the reporting methods used to present the research findings and describe the findings that the researcher has interpreted from the themes that emerged.

### Ethical consideration

Ethics approval was granted by the University of Pretoria, Faculty of Health Sciences Research Ethics Committee (423/2016), as well as the National Health Research Data Base, North West Provincial Office for Research, Monitoring and Planning.

## Results

**FIGURE 3:**
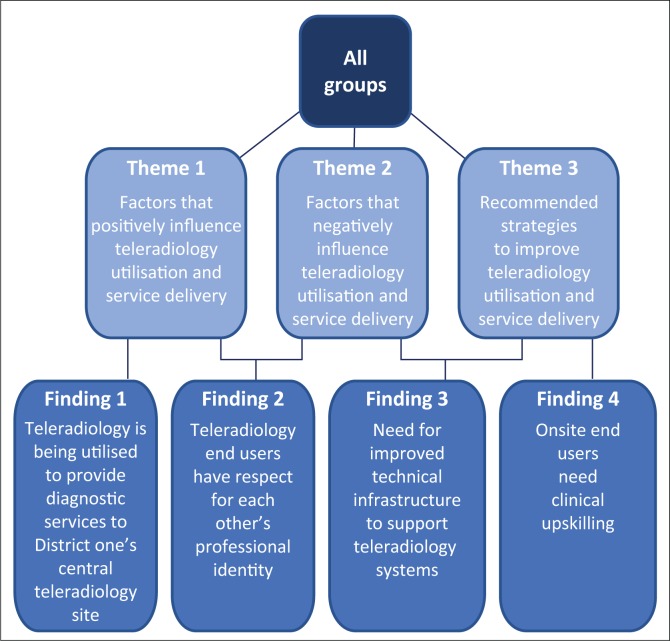
Findings from all participants emanating from three major themes.

The research findings were derived from the following themes shown in Figure 3.

The need for clinical upskilling of onsite end users

Findings 1–3 from this study (Figure 3) have previously been experienced by end users both locally and internationally.^7,20,21^ In this study, finding 4 seems to be unique, namely, the need for clinical upskilling of onsite end users. The views of the different end users are described in the following text.

### Radiographers

As described in Section 1, referring clinicians first come into contact with radiographers rather than the radiologist. Radiographers have an extended role beyond image acquisition, which includes authorising radiological requests and deciding on a scanning protocol based on clinical indications. Some radiographers expressed a lack of confidence in engaging with referring clinicians, as well as making decisions. Radiographers attributed their lack of confidence to not having advanced CT training. The following narratives were derived from FDG 1:

‘We don’t ask questions [*to the referring clinician*], we just book (the requested examination).’ (Rad 4)

‘… I don’t think I can make these decisions, because I have not had CT training, I only learnt CT when I came here.’ (Rad 1)

In this study, radiographers were unlikely to have postgraduate training in CT, which may explain their lack of confidence in engaging with referring clinicians and seeking the mandatory justification for the requested radiological examinations. When reflecting on other end users, radiologists suggested that junior referring clinicians appeared to request specialised imaging because the modality was available and not because the patient needed the investigation:

‘The patient becomes a guinea pig, we get a lot of interns, for them it’s a matter of experiencing.’ (Rad 2)

### Referring clinicians

Similar to radiographers, referring clinicians were also expected to take on roles traditionally performed by the radiologist, specifically in the CT department. These roles included injecting contrast media and responding to contrast-induced allergic reactions. The researcher asked the referring clinicians what their experiences were in this setting. Two junior referring clinicians expressed a lack of confidence and knowledge. The following narratives were derived from FGD 2:

‘Well to be honest, I have never been prepared for CT scan.’ (Phys 10)

‘I have been called [*by the radiographer to inject contrast media*], but I wouldn’t know how to react if the patient reacts.’ (Phys 11)

Senior referring clinicians expressed concerns that not all radiographers could perform specialised CT examinations:

‘I experience problems getting CT angio (Angiogram), when it comes to CT Angio not all radiographers can do that, at times we have to wait for a certain radiographer to come.’

‘They (specialists at referral hospital) don’t accept that there is no one (trained radiographer), to do it (specialised radiological examination).’ (Phys 4)

Referring clinicians are not allowed to refer patients to specialised institutes for further management, without a complete diagnostic work-up, which negatively impacts service delivery.

### Radiologists

The radiologist indicated that they did not receive images of optimal quality from the central teleradiology site of district 1 and attributed this to radiographers not sending appropriate images, and incorrectly planned examinations. The following narrations were derived from the face-to-face interview with the radiologist:

‘The images are not adequate, it’s sent in only an axial, its either missing the sagittal or coronal views, lung windows and that, so when it’s done in the peripheral site (central teleradiology site), it’s very difficult for me to manipulate on this side, so that’s the biggest challenge I have.’ (RS 1)

The radiologist also found reporting challenging because of not receiving sufficient clinical histories from radiographers and referring clinicians:

‘Sometimes all we get is a technote from the technician (radiographer) which is not adequate, the problem is twofold, it’s from the technotes and from the clinicians.’ (RS 1)

Onsite end users stated that radiologists did not have any onsite training needs because they were specialists. Radiographers mentioned that radiologists did not collaborate effectively and did not understand the complex context in which radiographers were practicing. The following narratives were derived from FGD 1 and 2, respectively:

‘we really don’t know who to contact for advice. I just feel if they (radiologist) could give us support.’ (Rad 4)

In this study, complex requests, particularly regarding paediatric patients, would be preceded by radiographers requesting that referring clinicians consult with radiologists before performing the scan. In such cases, referring clinicians felt unsupported by radiologists:

‘they (radiologist) say you are sitting with the patient there, what is your clinical judgement? you don’t have to call for all of them. It was the neurologist who requested the CT scan not us (referring clinician), so when I explain that, they (radiologist) get upset.’ (Phys 6)

## Discussion

From the views of the onsite end users, it is evident that both radiographers and referring clinicians were taking on roles that were traditionally performed by the radiologist. In this study, the SLA clearly outlines the roles and responsibilities of the end users. Radiographers must always produce quality images, provide timeous distribution of reports and monitor turnaround times. Referring clinicians, referred to as physicians, must ensure that the examination is necessary and adequate, and should always provide a clear history. Radiologists are responsible for justifying the examination dependent on the components of each case and should indicate if the routine protocol requires any modification, and the correct type and the amount of contrast to be injected.^9^ In contrast to the above-described roles, the participants in this study described performing tasks not described in the SLA. If the end users were to limit their practice to the SLA, the teleradiology operation would be dysfunctional and service delivery may be compromised. To avoid this, end users should be provided with the necessary support and training to fulfil these extended roles.

### The need for radiographers to be upskilled

From the interviews, referring clinicians expressed the concern that radiographers were unable to perform specialised imaging. Andronikou^22^ also described teleradiology being limited by poor-quality images because of radiologists being at a distant location and having little influence over the radiographer performing the radiological examination. Swensen and Johnson^23^ explain that the quality of radiological examinations can be improved with adequate protocol selection and standardisation of best practices. Swensen and Johnson further elaborate that radiologists should decide how to perform examinations and should guide the radiographer. In our study, radiographers had to take on the role of the radiologist, including justifying the request and deciding on protocols.^23^

### The need for referring clinicians to be upskilled

In this study, both the radiographers and the referring clinicians themselves felt that clinicians had inadequate competency in the CT department. Radiographers expressed concerns over inappropriate examination requests from clinicians. This could be because there were no guidelines for medical imaging among the 30 primary healthcare guidelines for primary clinicians.^24^ Ramanathan^25^ describes that clinicians need to enhance their knowledge of CT radiation doses to avoid requests for unwarranted scans. With regard to contrast media, referring clinicians were uncomfortable with having to inject contrast media, and shared their fear of not knowing how to react to a contrast-induced anaphylactic reaction. According to the SLA, injecting contrast media is beyond the referring clinician’s scope of practice.

### Computerised tomography training

In South Africa, teleradiology is used to transmit mostly CT to radiologists, from rural areas.^7,20,26^ Computerised tomography is a specialised field in radiology but is incorporated in the undergraduate radiography programme in South Africa. This undergraduate training does not adequately prepare radiographers to operate independently in a teleradiology setting, and their knowledge of CT applications should be augmented to perform CT examinations safely and accurately. Currently, there are limited postgraduate education and training opportunities available in South Africa, with only one academic institution formally offering the course as an elective.

### Teleradiology training

Teleradiology training is proposed to improve the skills of rural healthcare professionals operating in primary healthcare settings that use eHealth systems. The South African eHealth strategy document lists universities in the KwaZulu-Natal, Eastern Cape and Western Cape that offer introductory telemedicine training programmes to develop competent and skilled telemedicine practitioners.^27^ These courses cater to medical practitioners in the context of telemedicine but do not cater for teleradiology specifically.^27^ In South Africa, central sites, including academic institutes, are located as far as 1000 km away from the central and northern parts of South Africa, which have many vulnerable rural communities who depend on eHealth systems. Even though there is great demand, economical constraints prevent the DoH from training large groups of health professionals.^28^

### Recommendations

Given the unique findings of this study, we recommend that the roles of end users described in the SLA be revised and aligned with current teleradiology needs. Once established, end users should be trained to support their extended roles. Radiographers should be trained to plan examinations and evaluate the appropriateness of examinations. Referring clinicians should be trained to enhance their knowledge of the appropriate requesting pathways, risks of ionising radiation in CT, as well as the importance of a comprehensive clinical history. Radiologists should be introduced to the concept of inter-professional collaboration in a primary healthcare setting.

### Limitations

The findings from this study are unique to this primary healthcare setting and cannot be generalised to other settings. We recommend that other studies be conducted in teleradiology settings to identify unique challenges. At the time of data collection, only one radiologist, from a workforce of three, was available for an interview. Because of limited radiologists and high workload, we anticipated poor attendance of focus group interviews. In future, we recommend developing a short questionnaire be developed for radiologists.

## Conclusion

Teleradiology SLAs should be aligned to the clinical needs of the district. The SLA should guide the specific training needs of onsite end users. These training needs cannot be blanketed under eHealth training, which is suggested by the eHealth strategy document. Educational opportunities should be consistent and end user centred, with audited outcomes to ensure that programmes are effective. We envision that upskilling of individual end users will complement the extended roles in the teleradiology setting and will enhance the multidisciplinary approach to improve service delivery in the primary healthcare setting.
